# A Protest against Open Faced Crowns

**Published:** 1904-06

**Authors:** Frederick C. Royce


### A PROTEST AGAINST OPEN FACED CROWNS.

By Frederick C. Royce, D. D. S.

Read before the Second District Dental Society, November, 1903.

In the construction of a bridge where one of the anterior teeth is to be used as a support, we are confronted with the question, "What kind of a crown shall be used?" There are two important points to be considered, namely, usefulness and appearance; as we not only want a crown that looks well in the mouth but one that is also serviceable. We all dislike very much to see gold crowns on any of the anterior teeth, as they are anything but æsthetic in appearance, and, of course, would not use one, at least not for our lady patients. In the mouth of a man who has a moustache the all gold crown might be used and not be very noticeable.

There are many who object to the removal of the pulp to make use of a Richmond crown for an abutment and not being willing to use an all gold crown resort to the open face crown. Many dentists who are skilful mechanics make use of this style of crown, still I have no doubt that we all have seen cases where the use of them has resulted in failures. It is claimed by those who use this method of crowning that if a perfect adaptation of the gold to the shape of the tooth is obtained and the edges are thoroughly burnished down that the cement will not wash out. Theories may be advanced and new methods introduced for which much is claimed, but clinical experience is the only true test. From my experience I am firmly convinced that it is not possible to make an open faced crown, the cement of which will not be acted upon in the course of time by the fluids of the mouth.

In the preparation of a tooth to receive a crown it must be ground sufficiently to allow the band to slip over the tooth in order to fit at the neck. This will of necessity give a large surface that will be much more rapidly acted upon by decay.

After the cement is gone, sooner or later decay will begin under the gold, and it works so insidiously that suddenly the patient awakens to the fact that there is trouble with the tooth thus crowned. Upon examination it will be found that decay has begun and it is necessary to remove the crown. Perhaps at the end of the bridge is a full gold crown which it is necessary to slit open and pry off before the piece can be removed, and then what a sorry sight is presented. The tooth under the full gold crown is probably in as good condition as when crowned, while decay has taken place under the open faced crown until possibly the pulp is nearly or quite exposed. Not only that, but as a rule the dentine in such conditions is so very sensitive that it is almost impossible to work on it, even after having used obtundents. Now what shall be done with such a case as this? Perhaps it is decided to devitalize the pulp and use a Richmond crown, but upon a closer examination it is found that decay has extended above the gum margin either on the labial or lingual surface.

This may seem like an exaggerated story, but it is simply a statement of what has taken place in many months where this method of crowning has been used. Had the pulp been devitalized in the first place the bridge might have been anchored with a Richmond crown and the root prepared in just the shape the operator desired and the result have been a permanent piece of work. With the surface of decay extending up under the gum margin it is far more difficult to prepare the root and adjust the band to make an æsthetic as well as a lasting piece of work.

I have given open faced crowns a fair test, making them over metal dies, using 22k. gold, annealing well and burnishing the edges as close to the tooth as possible. The result with me was always the same; as a permanent piece of work it was a failure and in every case the bridge had to be removed and an anchorage made in some other way.

I have been called upon to remove bridges where the trouble was solely with open faced crowns, the anchorage at the other end being a full gold crown always found in perfect condition.

The workmanship in all the cases as far as I could see was good and satisfied me that they had come from the hands of good operators.

Now why are all these failures? It can not be that it is the fault of the operators in all these cases, as their other styles of crowns were doing splendid service and the failure was always with the open faced crowns. There is only one conclusion that can be drawn in the matter and that is, the method is at fault.

